# Immunology and Immunotherapy of Endometriosis

**DOI:** 10.3390/jcm10245879

**Published:** 2021-12-15

**Authors:** Radosław B. Maksym, Marta Hoffmann-Młodzianowska, Milena Skibińska, Michał Rabijewski, Andrzej Mackiewicz, Claudine Kieda

**Affiliations:** 1 Department of Reproductive Health, Centre of Postgraduate Medical Education, 01-004 Warsaw, Poland; mirab@cmkp.edu.pl; 2 Laboratory of Molecular Oncology and Innovative Therapies, Department of Oncology, Military Institute of Medicine, 04-141 Warsaw, Poland; mhoffmann-mlodzianow@wim.mil.pl (M.H.-M.); claudine.kieda@cnrs-orleans.fr (C.K.); 3 Doctoral Studies, Medical University of Lodz, 90-419 Lodz, Poland; milena.skibinska@gmail.com; 4 Department of Medical Biotechnology, Poznan University of Medical Sciences, 61-806 Poznan, Poland; a.mackiewicz@ump.edu.pl; 5 Department of Diagnostics & Cancer Immunology, Greater Poland Cancer Centre, 61-866 Poznań, Poland; 6 Centre for Molecular Biophysics, UPR CNRS 4301, CEDEX 2, 45071 Orléans, France

**Keywords:** endometriosis, immunotherapy, hypoxia, infertility, vaccine, ethiodized oil, NK cells

## Abstract

Endometriosis is one of the most common gynecological and systemic diseases, with a remarkable immune background. Patients suffer from pain and fertility reduction. Due to the distinct immune component, an immunotherapeutic approach may gain importance in the future. In endometriosis, shifts in the cell fractions of the immune system are well known. Moreover, hypoxia concomitant with inflammation causes a disturbed immune response. The removal of endometriosis has a therapeutic effect, normalizes the immune disorders, and remains the most effective causative treatment in terms of pain and infertility. A key issue is whether a similar effect can be achieved for fertility with non-invasive immunotherapy where surgery is inadvisable or cannot be performed for various reasons. Numerous immunotherapy trials, including vaccines, were conducted on animals only, although the research is encouraging. Among the promising methods of non-specific immunotherapy is the administration of an ethiodized oil contrast. Moreover, due to the significant successes of immunotherapy in oncology, the possibility of immunotherapy affecting NK cells has been postulated. NK cells are responsible for the surveillance and apoptosis of ectopic cells. Expanding the arsenal of endometriosis treatment by immunotherapy is promising due to the significant contribution of immunological factors and the limitations of current treatment methods.

## 1. Endometriosis as an Immunological Condition

Endometriosis is a chronic and inflammatory gynecological disorder in women of reproductive age. Affecting approximately 2–10% of women of childbearing age, it can extend symptoms and complications to the postmenopausal period [1]. Annual morbidity has been estimated at 1–3 per 1000 women [2,3]. Despite the publication of many studies, the pathogenesis of endometriosis has still not been fully explained. It is believed that the development of endometriosis is influenced by both hereditary factors and the effect of the environment, as well as by processes secondarily induced by endometriosis lesions. Notwithstanding the multifactorial causes, it is believed that the appearance of ectopic endometrial implants in the peritoneal cavity is possible due to immunological surveillance disturbances that allow for the survival of abnormal tissues which would otherwise be naturally eliminated. The presence of implants promotes oxidative stress and the gradual development of inflammation, while the macrophages present in the peritoneum produce growth and angiogenesis factors as well as inflammatory cytokines, which may be responsible for maintaining the disorder and the impairment of reproductive functions [4,5].

Endometriosis manifests itself as various forms of ectopic lesions on the peritoneum and ovarian endometrioma. A separate entity is deep endometriosis that infiltrates the tissues below the peritoneum, invading and functionally disturbing the bowel, ureters, bladder, nerves, uterosacral ligaments, and the rectovaginal septum.

**Figure 1:**
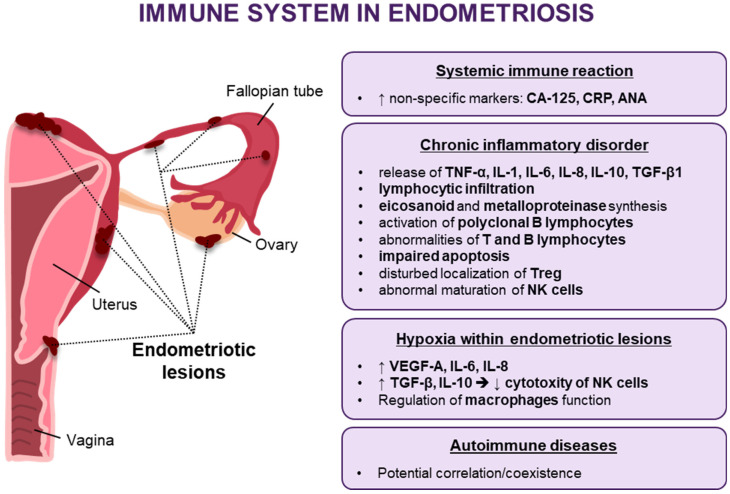
Dysregulation of the immune response in endometriosis (↑—increased, ↓—decreased, →—lead to).

Another closely related disease is adenomyosis, a kind of internal endometriosis characterized by an in-growth of the endometrial glands into the uterine muscle. It is often concomitant with other endometriosis forms, significantly affecting fertility. It is postulated that endometriosis and adenomyosis share a common etiopathogenesis. The uterus is formed through the fusion of the Müllerian ducts into a single tube to form the area of archimyometrium—the inner myometrium of the uterus, which plays an essential part in the etiopathogenesis of both adenomyosis and endometriosis [6]. The fusion of the paired ducts results in the formation of a fundocornual raphe, which is later subject to high stresses, leading to tissue damage during uterine peristalsis. and may contribute to fertility disorders in endometriosis [7,8]. It is worth emphasizing that despite common mechanisms and similar disorders related to reduced fertility in endometriosis and adenomyosis, and the frequent coexistence of these diseases, the treatment cannot be similar. In patients treated for infertility and adenomyosis, the surgical removal of these abnormal infiltrates is not possible as it would mean damage to the uterus. All the more urgent is the need to develop new non-invasive methods targeting common and still not fully understood disease mechanisms. Endometriosis forms a specific hormonal environment characterized by high concentrations of estrogens and androgens, whose levels are several times greater than those found in patients’ peripheral blood. Consequently, different phenomena are triggered, including cell proliferation, the release of various immunological and inflammatory factors, such as TNF, IL-1, IL-6, IL-8, IL-10, TGF-β1 cytokines, lymphocytic infiltration, or eicosanoid and metalloproteinase activation (see Figure 1) [9,10,11].

There is no consensus on whether each of the forms mentioned above progresses into the next one, whether they have a common pathogenesis, or whether one type of endometriosis may cause a different one. It is believed that the cause of deep endometriosis is rather embryological; therefore, the immune system would be of less importance in its development [12]. Superficial endometriosis and ovarian endometriomas are usually considered as the effects of the implantation of menstrual debris containing living clusters of endometrial cells. These cells transfer to the peritoneal cavity via antiperistaltic flow through the oviducts, called retrograde menstruation [13], which can be enhanced by cervical stenosis or improper uterine constrictions [6,14,15]. Since it is well known that the majority of healthy women have retrograde menstruation and this does not cause endometriosis, other pivotal factors have to play a role [16]. An altered immune reaction is the main factor considered responsible for deregulated immune surveillance, leading to the development of endometriosis and possible consequences, including infertility [17].

Therefore, endometriosis is considered a chronic inflammatory disease in which the inflammation is not limited to the peritoneal cavity alone. Immune reactions spread to the systemic level, as evidenced by elevated levels of non-specific markers such as CA-125 and CRP or the presence of antinuclear antibodies (ANA) in the serum [18,19,20]. Endometriosis thus meets many of the classification criteria for an immune disorder. Numerous immune disorders indicate pathogenetic mechanisms and potential treatment options. Endometriosis is characterized by polyclonal lymphocyte B activation, lymphocyte T(Th1) and B dysfunction, impaired apoptosis, and NK cell activity. In some patients, NK cell cytotoxic activity among the endometrial cells is abnormal [21]. A Th1/Th2 equilibrium shift in the stroma of endometriotic lesions and on the systemic level towards a Th2-dependent response has also been described [22,23]. Immune deregulation can be associated with the described translocation of T regulatory (Treg) cells [24].

**Figure 2:**
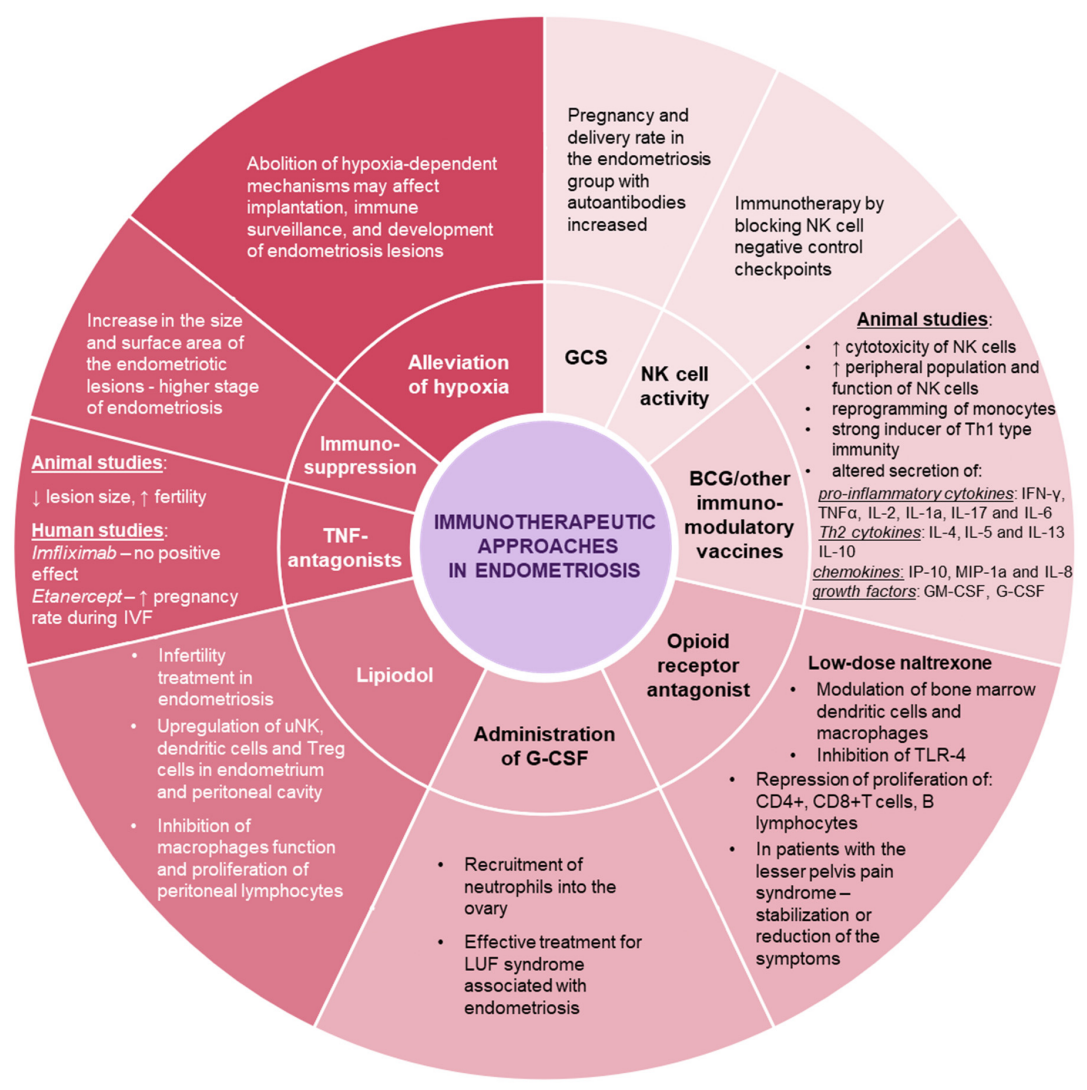
Schematic representation of the therapeutic approaches that can modulate the immune system in endometriosis (↑—increased, ↓—decreased).

There is a significantly increased incidence of immune-related disorders in patients with endometriosis, particularly autoimmune diseases and celiac disease, which are often latent, or a genetic predisposition to the disease. The coexistence of endometriosis with systemic lupus, Sjögren’s syndrome, rheumatoid arthritis, autoimmune thyroiditis, multiple sclerosis, Addison’s disease, and inflammatory bowel disease has been proven to be many times greater. Although there are no convincing data on a possible causal mechanism linking these pathologies with endometriosis or on a common cause underlying these disorders, it is postulated that impaired immune regulation is a combined substratum of endometriosis and autoimmune diseases [21]. Moreover, concomitant autoimmunity is associated with a more severe course of endometriosis [18]. Endometriosis patients suffer from celiac disease three times more often than healthy women. Therefore, a gluten-free diet eliminates additional pro-inflammatory stimulation in susceptible patients [25]. Due to the remarkable immune background of the disorders present in endometriosis and associated conditions we postulate, that immunotherapy may constitute promising approach and a useful direction in the treatment of this condition. Moreover, immunotherapy may prove to be the first effective non-invasive treatment of endometriosis-related infertility, as standard hormonal drugs do not improve fertility and cannot be used in patients trying to become pregnant [1]. The therapeutic modalities for endometriosis, as well as, endometriosis-associated infertility, that are discussed in this paper, are briefly summarized in Figure 2.

## 2. Hypoxia-Dependent Development of Endometriosis

Retrograded endometrial tissues need to attach to the surfaces of organs in the peritoneal cavity and implant in order to survive ectopically. Hypoxic stress and adhesive ability are two challenges that the cells must face. The most common method to determine tissue hypoxia is the detection of the oxygen sensing hypoxia inducible factor 1α (HIF-1α) which is best described effector and plays a key role in cellular response to low oxygen levels [26]. In endometriosis, it was found that ectopic lesions demonstrate an increased expression of the HIF-1α monomeric isoform, which is a hallmark of hypoxia, in comparison to a eutopic endometrium [27]. Additionally, the inhibition of HIF-1α production in a mouse model of endometriosis suppressed the growth of lesions, indicating hypoxia as a potential therapeutic target [28]. It has already been shown that endometriotic tissues in women present increased levels of hypoxia that induce the expression of numerous important downstream genes to stimulate the establishment of ectopic lesions via the enhancement of adhesions, angiogenesis, and proliferation [29,30,31,32]. Two recent studies have shown that hypoxia enhances the cell adhesive ability of endometrial stromal cells by inducing the expression of cell adhesion molecules [30,33]. In addition, the active neovascularization of endometrial lesions involves proangiogenic factors, the most prominent of which is vascular endothelial growth factor (VEGF-A), the expression of which is stimulated under hypoxic conditions in response to the HIF-1 transcription pathway [34]. Chronic inflammation in endometriosis is also a strong factor in angiogenesis development. IL-1β, tumor necrosis factor (TNF), transforming growth factor β (TGF-β), and IL-8 are strong inducers of angiogenesis in endometriotic tissues. Nevertheless, hypoxia was demonstrated to be the upstream factor, which upregulates VEGF-A, IL-6, and IL-8 in endometriosis [35,36,37]. Research data have shown that hypoxia regulates and induces several key genes and mechanisms during and after the implantation of endometriotic lesions. Moreover, some of these mechanisms actively interact and modulate the specific type and activity of immune cells. For instance, TGF-β, the expression of which is stimulated by hypoxia and, thus, elevated in the peritoneal fluid from patients with endometriosis, was related to the reduced cytotoxicity of NK cells in endometriosis [30,38,39]. A study by Yang et al. showed that IL-10 and TGF-β derived from the co-culture of endometrial stromal cells and macrophages may be responsible for reduced killing-associated cytokine secretion as well as for the reduced cytotoxicity of NK cells in women with endometriosis [39]. In addition, peritoneal macrophages highly infiltrate endometriotic implants and hypoxia was described as a master modulator of macrophage function in endometriosis [40]. These findings are in line with a study by Thiruchelvam et al., who reported that an abnormal maturation of NK cells occurs in endometriosis; thus, they hypothesize that the cytotoxicity of NK cells, isolated from the ectopic endometrium, is lower than of those from the eutopic endometrium and healthy controls [41]. In addition to this indirect impact of hypoxia, its direct action on the NK cells was observed in tumor sites. Parodi et al. showed that a hypoxic environment may profoundly influence the type and function of NK cells reaching hypoxic tissues and impact the immune-mediated responses within tumor tissues [34]. Moreover, it was shown that NK cell infiltration and its antitumor activity are impaired by the immune checkpoint molecule PD-L1, which is expressed under hypoxic conditions on endothelial cells in the tumor. The normalization of a hypoxic condition reverts immunosuppression into an antitumor immune response [42]. We may thus hypothesize that the same type of mechanism occurs in endometriosis lesions; nevertheless, further investigation is needed.

The presented data point out that hypoxia is one of the crucial factors in the development of endometriosis in women. It has also been noticed that hypoxia influences other processes and particularly affects the efficacy of the immune response. Hence, considering the prominent development of immunotherapy, the design of immunotherapeutic strategies should take into account the context of hypoxic microenvironment [34]. Thus, the alleviation of hypoxia as a concomitant treatment to immunotherapy in endometriosis treatments may provide new approaches to control the disease in the future. The clinical trials conducted so far on myo-inositol trispyrophosphate (ITPP) as a compound decreasing the level of hypoxia have shown that there is no apparent toxicity and this type of therapy is a well-tolerated adjunct to oncological treatment [43,44].

## 3. Therapeutic Approaches to Endometriosis

### 3.1. Surgical Removal of Ectopic Lesions

The surgical removal of endometriosis lesions is a method of treatment with proven effectiveness in reducing pain and improving fertility in patients with endometriosis. Although one of the most effective forms of treating pain in endometriosis is hormonal treatment, it cannot be used in patients trying to become pregnant because it does not improve the effect of efforts at any stage of treatment [1]. A thorough discussion of endocrine therapy is beyond the scope of this manuscript, which mainly focuses on endometriosis in the context of fertility disorders. It has been known for a long time that the destruction of lesions leads to the alleviation of immune disorders. The removal of the ectopic foci, which release immunosuppressive agents and impair the ability of NK cells to eliminate endometrial cells from the peritoneal cavity, reduces their negative impact [45]. Thus, the complete eradication of endometriotic lesions should be considered as the most effective form of causative treatment that also affects the immune system and offers high potential for improving the pregnancy rate. The probability of spontaneous conception is represented by the endometriosis fertility index (EFI) that is calculated in post-operative patients. Up to 74.9% ± 4.2% of patients with the best prognoses conceive spontaneously within three years following the radical removal of lesions. The stage of endometriosis is of lesser importance in such cases. Factors that are taken into account include age, how long the patient has been trying to conceive, any previous pregnancies, and the condition of the reproductive organs after the endometriosis surgery [46].

### 3.2. Immunosuppressive Treatment

Except for observational, cross-sectional studies, it is difficult to perform reliable investigations on endometriosis patients. The majority of immunological endometriosis research is performed on animals. In nature, spontaneous endometriosis affects only those mammalian species that menstruate, including: primates, some bat species, the spiny mouse and elephant shrew [47]. Many animal models are not fully reliable, since endometriosis is induced artificially and does not represent all the phenomena present in the disorder. Generally, with various technical modifications, endometrial tissue (sometimes along with fragments of the myometrium) originating from the uterine cavity is translocated into ectopic locations. The development of this tissue is then intended to represent the various processes that occur during the development of endometriosis. The entire model is also disturbed by the necessity to undergo procedure, which may also lead to significant injury and inflammatory or infectious stress. Other models that must utilize primates are expensive and troublesome for many reasons. Moreover, artificial endometriosis behaves differently than spontaneous endometriosis, even in the same experimental animal model or in the same animal [48,49].

In order to verify the influence of the immune system and the possible role of immunosuppression on endometriosis, an animal model was implemented despite the above reservations. Olive baboons develop endometriosis spontaneously; thus, they are a better model for tracking the course of the disease. As early as in 1995, D’ Hooghe treated female olive baboons with immunosuppressive therapy combined with methylprednisolone and azathioprine—similar doses to the pharmacotherapy after an allogeneic heart transplant. Female baboons with spontaneous endometriosis showed an increase in the size and surface area of the lesions and a more advanced stage of the disease according to the American Fertility Society (AFS) classification. Newly developed ovarian endometrioid cysts were the result of azathioprine-only treatment. After immunosuppression discontinuation, at least a partial remission of the lesions was observed. At the beginning of the experiment, healthy baboons were subjected to the therapy and remained endometriosis-free. Moreover, surgically induced lesions failed to respond to immunosuppression [50]. This study was a significant warning and indicated that the indiscriminate and arbitrary use of immunological treatment, previously eagerly proposed to patients with infertility or habitual miscarriages, may abolish immune surveillance, paradoxically aggravate the course of endometriosis and worsen the effects of infertility treatment.

### 3.3. Glucocorticosteroids

Also worth mentioning are the outcomes on short-term immunotherapy using glucocorticosteroids (GCS), derived from the study applying the method of in vitro fertilization (IVF) in patients with endometriosis and patients with a tubal factor of infertility. In patients with endometriosis, the presence of autoantibodies was significantly more prevalent than in healthy controls [18,19] and patients with tubal factors only. Researchers have proven that transient prednisolone administration from the 3rd day of the menstrual cycle significantly raised the pregnancy rate in the endometriosis group with antibodies. After the establishment of pregnancy, steroid therapy was not prolonged and endometriosis was not found to further influence the course of the pregnancy [51]. Another study showed that steroid use during an IVF cycle in autoantibody positive patients was associated with a higher clinical pregnancy rate (80% vs. 0%, *p* < 0.05); however, the effect of steroids in autoantibody-negative patients was not beneficial (46.7% vs. 45%, *p* = NS) [20].

### 3.4. TNF-Antagonists

A positive effect of drugs inhibiting the action of TNF on the development of endometriosis has been shown in animal models. There was a reduction in lesion size and an improvement in the fertility indicators, such as apoptosis and embryotoxicity. However, the administration of the TNF antagonist, imfliximab, in women with endometriosis did not reduce the pain symptoms or the size of the lesions. Its positive effect on fertility has also not been analyzed. Etanercept, another TNF antagonist, has been shown to have a positive effect on pregnancy rates in patients with endometriosis-associated infertility or ovarian endometrial cysts undergoing IVF treatment [52,53]. The data on the use of etanercept in other diseases indicate its high safety in early pregnancy. Unfortunately, the available data are scarce and do not allow this type of treatment to be recommended for routine usage at the moment [10].

### 3.5. Vaccines

Another postulated treatment proposition involves the Bacillus Calmette–Guérin (BCG) tuberculosis vaccine—one of the most effective immunotherapeutic agents with long application experience. It has been successfully used in infectious diseases and oncology for a long time and may find yet another application in endometriosis therapy. BCG administration triggers NK cells and induces factor recruitment, increasing the cytotoxicity of the NK cells. Animal studies have confirmed that subjects previously treated with the BCG vaccine had significantly endometriosis lesions [54]. BCG vaccination alters the secreted pro-inflammatory cytokines, IFN-γ, TNF, IL-2, IL-1a, IL-17 and IL-6; the Th2 cytokines, IL-4, IL-5, IL-13, and IL-10; and the chemokines, IP-10, MIP-1a, and IL-8, as well as the growth factors, granulocyte macrophage colony-stimulating factor (GM-CSF) and granulocyte colony-stimulating factor (G-CSF). Moreover, BCG upregulates the peripheral population and the function of NK cells, and also reprograms monocytes. As mentioned above, a polarization towards a Th2 response has been observed in endometriosis [23]. BCG is a strong inducer of Th1-type immunity and has been reported to protect against Th2-driven disorders. Despite promising opportunities, there is still a lack of data to demonstrate the effectiveness of this strategy in the human model [55]. There have also been attempts to develop and apply more specific vaccines with an immunotherapeutic effect in endometriosis. Studies with the use of a vaccine imitating the antigens of endometriosis and uterine fibroids seem to be interesting. In an animal model, a significant impact of administering this type of experimental vaccine on the subsequent possibility of induction of endometriotic foci has been demonstrated. In these immune-protected rats, the probability of the induction of endometriosis decreased from 69.6% to only 4.3% [56]. The administration of the vaccine before the induction of endometriosis prevented the increase of Th-2 dependent cytokines: IL4, IL6, and IL-10, which is typically observed during the development of endometriosis [57].

### 3.6. NK Cells Modulation

The central role of NK cells in the immune dysregulation exerted by endometriotic implants, as well as the rapid normalization of their activity after endometriosis removal, is discussed above [45]. Therefore, the possible application of immunotherapy to influence NK cell activity has also been suggested. NK cells have inhibitor receptors on their surface that suppress their activity against ectopic or malignant cells. Among receptors, PD1 that binds to the PDL1 ligand has already successfully been implemented in cancer immunotherapy. This kind of treatment may attempt to influence endometrial cell rescue, abolish the suppression of the supervisory function of NK cells, and allow for the removal of ectopic endometrial cells. The suggested immunotherapy postulates the application of medicines already used to treat other conditions, such as cancers, to facilitate the detection and elimination of endometrial cells through apoptosis [58].

### 3.7. Endorphin Modulation

Another potentially helpful agent in endometriosis treatment is low-dose naltrexone (LDN). As an opioid receptor antagonist, it has long been used in addiction therapy, chronic pain syndromes, and autoimmune diseases. The administration of low doses, as opposed to high doses, stimulates endorphin release and allows for immune system modulation by the central nervous system. Among other effects, LDN modulates the function of immune cells, such as bone marrow dendritic cells and macrophages, by methionine enkephalin (OGF), a potent antitumor agent, as well as by the inhibition of Tool-like receptor 4 (TLR-4) [59]. Moreover, LDN represses the proliferation of CD4+ and CD8+T cells and B lymphocytes in the spleen and lymph nodes [60]. The drug exerts slight transient side effects in 5% of patients, including sleep disturbances, daydreaming, nausea, headaches, and mucosal dryness. Naltrexone increases the efficacy of ovulation stimulation by anti-estrogens—being a cheaper and safer application than gonadotropins. An analysis of naltrexone use in polycystic ovary syndrome revealed a statistically significant decrease in BMI and insulin, LH/FSH, and testosterone levels in patients treated with naltrexone, which resulted in a 33% pregnancy rate. In the majority of patients with pelvic pain syndrome, LDN therapy allowed at least a stabilization or reduction in symptoms without compromising fertility [61,62].

### 3.8. G-CSF

A common problem in endometriosis patients that could be an additional reason for infertility is recurrent luteinized unruptured follicle syndrome (LUF), which affects up to 30% of menstrual cycles in endometriosis patients, compared to 6% in healthy women. Particularly in patients with ovarian endometriomas, LUF may affect more than 50% of cycles [63]. The lack of proper ovulation in such cycles makes it impossible to become pregnant and if LUF occurs regularly, it may be the only cause of infertility in this group of patients with endometriosis. Interestingly, neutrophils appear to play an essential role in ovulation as they are required to release metalloproteinases and digest the sheath of the Graafian follicle. In the absence of proper stimulation by G-CSF, neutrophils cannot be appropriately recruited into the ovary, and the follicle cannot release the oocyte correctly. The significantly increased frequency of the LUF syndrome in endometriosis is explained by the dysregulation of the neutrophilic function resulting from an impaired immune response. A logical treatment for LUF seems to be the external administration of G-CSF. A study on a patient with recurrent LUF was conducted in which doses of 30–45 thousand units of G-CSF were administered 24–48 h before chorionic gonadotropin administration in a stimulated cycle. After each cycle during which G-CSF was incorporated into the stimulation protocol, regular ovulation occurred and the post-ovulatory phase was stable and unimpaired. Where no G-CSF was administered, ovulation was abnormal with functional cyst (LUF) formation. Up to 90% of patients with infertility ovulated after the administration of the stimulating factor, compared to only 50% of the controls [64]. Therefore, G-CSF appears to be a safe and effective treatment for LUF syndrome associated with endometriosis.

### 3.9. Ethiodized Oil Perfusion

Finally, it is worth pointing out a simple method of immunotherapy already used in practice, the detailed description and mechanisms of which go beyond the scope of this study. The immunotherapeutic effect in endometriosis-related infertility can be attributed to the intra-uterine administration of an ethiodized oil contrast agent, Lipiodol. A significant improvement in fertility, especially in patients with endometriosis, was noticed after the application of this agent, which was used primarily to assess the patency of the Fallopian tubes. The increase in fertility is estimated at 2 to 5 times within 6 months. Finally, in the three-year follow-up, more than half of the patients became pregnant, regardless of whether they were burdened with endometriosis or with so-called idiopathic infertility, which can also be associated with an undiagnosed immune background. This initially unintended effect was noticed after an analysis of the unexpectedly high pregnancy rates after patency examination if oil contrast was used. It was proven that Lipiodol upregulates uNK and dendritic and Treg cells in the endometrium, as well as in the peritoneal cavity. The application of an oil contrast also inhibits peritoneal lymphocyte and macrophage function. At the moment, however, it is not clear which mechanism plays the greatest role in improving fertility. Currently, oil flushing is becoming more and more widely used as an independent method of infertility treatment in endometriosis and idiopathic infertility [65,66,67,68].

## 4. Conclusions

Immunotherapy, as a tool in the treatment of endometriosis, remains a controversial issue. Currently, it is still not included in the guidelines or recommendations for endometriosis treatment [1,69,70,71]. However, it is believed that immunotherapy may become useful in the treatment of this condition in the future. At present, there are no methods of selective immunotherapy that can be used in routine clinical practice. According to the guidelines, in patients with symptoms of endometriosis, who are currently not trying to conceive, the first-line treatment is hormone therapy. If the pain is significant, pharmacotherapy proves ineffective; if fertility is one of the goals of treatment, laparoscopic surgery may be implemented [1,69,70,71]. Infertility in endometriosis results from damage to the anatomy (adhesions and nodular degeneration of the Fallopian tube isthmus), immune dysregulation (Th1/Th2 shifts, NK, Th17, and Treg cell activity disruption), gameto- and embryotoxicity, Fallopian ciliary dyskinesia, ovulatory and uterine peristalsis disorders, as well as coincidental adenomyosis, that cannot be treated surgically. The surgical removal of lesions improves fertility in patients treated for infertility. The excision or destruction of endometriosis lesions have a direct impact on immune normalization [45] and the subsequent fertility level can be calculated [46]. In cases where it is known that anatomy disorders are not the cause of infertility, and the involvement of an immune factor is suspected, a good solution seems to be the implementation of minimally invasive treatment, including an immunotherapeutic approach. Some methods, including Lipiodol flushing or G-CSF, are supported by numerous publications and extensive clinical evidence. Other methods involving the normalization of hypoxia, TNF antagonists, PD1/PDL1 antagonists and vaccinations appear to be very attractive and their future development seems to be an interesting approach. If conventional treatment produces no results or is contraindicated, an experienced consultant can propose a noncanonical therapy to an informed patient, preferably in a clinical trial. Accurate knowledge of the detailed mechanisms of action of individual therapy methods still requires consolidation. It is also unclear whether the methods are effective for all cases suffering from the spectrum of endometriosis and endometriosis-like disorders. Neither of the presented therapies can be used as the sole therapy or panacea for all patients. The available state of knowledge on this subject has been presented herein. Due to the limitations of animal research and the few human studies that have been carried out, current knowledge is still incomplete and sometimes ambiguous. Considering all the hopes and limitations associated with such a therapeutic approach, the prospect of research in this field seems very promising. Evidently, further methodologically reliable studies to assess the safety and effectiveness of immunotherapeutic methods are needed to fully consolidate their place in endometriosis treatment algorithms.

## Data Availability

Not applicable.

## References

[1] Dunselman G.A.J., Vermeulen N., Becker C., Calhaz-Jorge C., D’Hooghe T., De Bie B., Heikinheimo O., Horne A.W., Kiesel L., Nap A. (2014). ESHRE guideline: Management of women with endometriosis. Hum. Reprod..

[2] Gylfason J.T., Kristjansson K.A., Sverrisdottir G., Jonsdottir K., Rafnsson V., Geirsson R.T. (2010). Pelvic Endometriosis Diagnosed in an Entire Nation Over 20 Years. Am. J. Epidemiol..

[3] Missmer S.A., Hankinson S.E., Spiegelman D., Barbieri R.L., Marshall L.M., Hunter D.J. (2004). Incidence of Laparoscopically Confirmed Endometriosis by Demographic, Anthropometric, and Lifestyle Factors. Am. J. Epidemiol..

[4] Foster W.G. (2018). Hypoxia-induced autophagy, epithelial to mesenchymal transition, and invasion in the pathophysiology of endometriosis: A perspective. Biol. Reprod..

[5] Koninckx P.R., Ussia A., Adamyan L., Wattiez A., Gomel V., Martin D.C. (2019). Pathogenesis of endometriosis: The genetic/epigenetic theory. Fertil. Steril..

[6] Leyendecker G., Bilgicyildirim A., Inacker M., Stalf T., Huppert P., Mall G., Böttcher B., Wildt L. (2015). Adenomyosis and endometriosis. Re-visiting their association and further insights into the mechanisms of auto-traumatisation. An MRI study. Arch. Gynecol. Obstet..

[7] Kuijsters N.P.M., Methorst W.G., Kortenhorst M.S.Q., Rabotti C., Mischi M., Schoot B.C. (2017). Uterine peristalsis and fertility: Current knowledge and future perspectives: A review and meta-analysis. Reprod. Biomed. Online.

[8] Guo S.-W. (2020). The Pathogenesis of Adenomyosis vis-à-vis Endometriosis. J. Clin. Med..

[9] Gibson D., Simitsidellis I., Collins F., Saunders P.T.K. (2020). Androgens, oestrogens and endometrium: A fine balance between perfection and pathology. J. Endocrinol..

[10] Kolanska K., Alijotas-Reig J., Cohen J., Cheloufi M., Selleret L., D’Argent E., Kayem G., Valverde E.E., Fain O., Bornes M. (2021). Endometriosis with infertility: A comprehensive review on the role of immune deregulation and immunomodulation therapy. Am. J. Reprod. Immunol..

[11] Bałkowiec M., Maksym R.B., Włodarski P.K. (2018). The bimodal role of matrix metalloproteinases and their inhibitors in etiology and pathogenesis of endometriosis (Review). Mol. Med. Rep..

[12] Mendiola J., Sánchez-Ferrer M.L., Jiménez-Velázquez R., Cánovas-López L., Peñalver A.I.H., Corbalán-Biyang S., Carmona-Barnosi A., Prieto-Sánchez M.T., Nieto A., Torres-Cantero A.M. (2016). Endometriomas and deep infiltrating endometriosis in adulthood are strongly associated with anogenital distance, a biomarker for prenatal hormonal environment. Hum. Reprod..

[13] Sampson J.A. (1927). Peritoneal endometriosis due to the menstrual dissemination of endometrial tissue into the peritoneal cavity. Am. J. Obstet. Gynecol..

[14] Zondervan K.T., Becker C.M., Missmer S.A. (2020). Endometriosis. N. Engl. J. Med..

[15] Brosens I., Gargett C.E., Guo S.-W., Puttemans P., Gordts S., Brosens J., Benagiano G. (2016). Origins and Progression of Adolescent Endometriosis. Reprod. Sci..

[16] Halme J., Hammond M.G., Hulka J.F., Raj S.G., Talbert L.M. (1984). Retrograde menstruation in healthy women and in patients with endometriosis. Obstet. Gynecol..

[17] Dmowski W., Steele R.W., Baker G.F. (1981). Deficient cellular immunity in endometriosis. Am. J. Obstet. Gynecol..

[18] Stella V.V., Roberta V., Noemi S., Enrico P., Diana D., Jessica O., Patrizia R.-Q., Stefano F., Paola V., Massimo C. (2021). Concomitant autoimmunity may be a predictor of more severe stages of endometriosis. Sci. Rep..

[19] Dias J., De Oliveira R., Abrão M. (2006). Antinuclear antibodies and endometriosis. Int. J. Gynecol. Obstet..

[20] Dmowski W.P., Rana N., Michalowska J., Friberg J., Papierniak C., El-Roeiy A. (1995). The effect of endometriosis, its stage and activity, and of autoantibodies on in vitro fertilization and embryo transfer success rates. Fertil. Steril..

[21] Shigesi N., Kvaskoff M., Kirtley S., Feng Q., Fang H., Knight J.C., A Missmer S., Rahmioglu N., Zondervan K.T., Becker C.M. (2019). The association between endometriosis and autoimmune diseases: A systematic review and meta-analysis. Hum. Reprod. Updat..

[22] Antsiferova Y.S., Sotnikova N.Y., Posiseeva L.V., Shor A.L. (2005). Changes in the T-helper cytokine profile and in lymphocyte activation at the systemic and local levels in women with endometriosis. Fertil. Steril..

[23] Podgaec S., Abrao M.S., Dias J.A., Rizzo L.V., De Oliveira R.M., Baracat E.C. (2007). Endometriosis: An inflammatory disease with a Th2 immune response component. Hum. Reprod..

[24] Olkowska-Truchanowicz J., Bocian K., Maksym R.B., Białoszewska A., Włodarczyk D., Baranowski W., Ząbek J., Korczak-Kowalska G., Malejczyk J. (2013). CD4^+^ CD25^+^ FOXP3^+^ regulatory T cells in peripheral blood and peritoneal fluid of patients with endometriosis. Hum. Reprod..

[25] Santoro L., Campo S., D’Onofrio F., Gallo A., Covino M., Campo V., Palombini G., Santoliquido A., Gasbarrini G., Montalto M. (2014). Looking for Celiac Disease in Italian Women with Endometriosis: A Case Control Study. BioMed Res. Int..

[26] Semenza G.L. (2001). HIF-1 and mechanisms of hypoxia sensing. Curr. Opin. Cell Biol..

[27] Wu M.-H., Chen K.-F., Lin S.-C., Lgu C.-W., Tsai S.-J. (2007). Aberrant Expression of Leptin in Human Endometriotic Stromal Cells Is Induced by Elevated Levels of Hypoxia Inducible Factor-1α. Am. J. Pathol..

[28] Becker C.M., Rohwer N., Funakoshi T., Cramer T., Bernhardt W., Birsner A., Folkman J., D’Amato R.J. (2008). 2-Methoxyestradiol Inhibits Hypoxia-Inducible Factor-1α and Suppresses Growth of Lesions in a Mouse Model of Endometriosis. Am. J. Pathol..

[29] Laschke M., Giebels C., Menger M. (2011). Vasculogenesis: A new piece of the endometriosis puzzle. Hum. Reprod. Updat..

[30] Lin X., Dai Y., Xu W., Shi L., Jin X., Li C., Zhou F., Pan Y., Zhang Y., Lin X. (2018). Hypoxia Promotes Ectopic Adhesion Ability of Endometrial Stromal Cells via TGF-β1/Smad Signaling in Endometriosis. Endocrinology.

[31] Lu Z., Zhang W., Jiang S., Zou J., Li Y. (2014). Effect of oxygen tensions on the proliferation and angiogenesis of endometriosis heterograft in severe combined immunodeficiency mice. Fertil. Steril..

[32] Zhan L., Wang W., Zhang Y., Song E., Fan Y., Wei B. (2016). Hypoxia-inducible factor-1alpha: A promising therapeutic target in endometriosis. Biochimie.

[33] Lin S.-C., Lee H.-C., Hsu C.-T., Huang Y.-H., Li W.-N., Hsu P.-L., Wu M.-H., Tsai S.-J. (2019). Targeting Anthrax Toxin Receptor 2 Ameliorates Endometriosis Progression. Theranostics.

[34] Parodi M., Raggi F., Cangelosi D., Manzini C., Balsamo M., Blengio F., Eva A., Varesio L., Pietra G., Moretta L. (2018). Hypoxia Modifies the Transcriptome of Human NK Cells, Modulates Their Immunoregulatory Profile, and Influences NK Cell Subset Migration. Front. Immunol..

[35] Hsiao K.-Y., Chang N., Lin S.-C., Li Y.-H., Wu M.-H. (2014). Inhibition of dual specificity phosphatase-2 by hypoxia promotes interleukin-8-mediated angiogenesis in endometriosis. Hum. Reprod..

[36] Hsiao K.-Y., Chang N., Tsai J.-L., Lin S.-C., Tsai S.-J., Wu M.-H. (2017). Hypoxia-inhibited DUSP2 expression promotes IL-6/STAT3 signaling in endometriosis. Am. J. Reprod. Immunol..

[37] Sharkey A.M., Day K., McPherson A., Malik S., Licence D., Smith S.K., Charnock-Jones D.S. (2000). Vascular Endothelial Growth Factor Expression in Human Endometrium Is Regulated by Hypoxia 1. J. Clin. Endocrinol. Metab..

[38] Kupker W., Schultze-Mosgau A., Diedrich K. (1998). Paracrine changes in the peritoneal environment of women with endometriosis. Hum. Reprod. Updat..

[39] Yang H.-L., Zhou W.-J., Chang K.-K., Mei J., Huang L.-Q., Wang M.-Y., Meng Y., Ha S.-Y., Li D.-J., Li M.-Q. (2017). The crosstalk between endometrial stromal cells and macrophages impairs cytotoxicity of NK cells in endometriosis by secreting IL-10 and TGF-β. Reproduction.

[40] Lin Y.-J., Lai M.-D., Lei H.-Y., Wing L.-Y.C. (2006). Neutrophils and Macrophages Promote Angiogenesis in the Early Stage of Endometriosis in a Mouse Model. Endocrinology.

[41] Thiruchelvam U., Wingfield M., O’Farrelly C. (2016). Increased uNK Progenitor Cells in Women with Endometriosis and Infertility are Associated with Low Levels of Endometrial Stem Cell Factor. Am. J. Reprod. Immunol..

[42] El Hafny-Rahbi B., Brodaczewska K., Collet G., Majewska A., Klimkiewicz K., Delalande A., Grillon C., Kieda C. (2021). Tumour angiogenesis normalized by myo-inositol trispyrophosphate alleviates hypoxia in the microenvironment and promotes antitumor immune response. J. Cell. Mol. Med..

[43] Limani P., Linecker M., Kron P., Samaras P., Pestalozzi B., Stupp R., Jetter A., Dutkowski P., Müllhaupt B., Schlegel A. (2016). Development of OXY111A, a novel hypoxia-modifier as a potential antitumor agent in patients with hepato-pancreato-biliary neoplasms-Protocol of a first Ib/IIa clinical trial. BMC Cancer.

[44] Schneider M.A., Linecker M., Fritsch R., Muehlematter U.J., Stocker D., Pestalozzi B., Samaras P., Jetter A., Kron P., Petrowsky H. (2021). Phase Ib dose-escalation study of the hypoxia-modifier Myo-inositol trispyrophosphate in patients with hepatopancreatobiliary tumors. Nat. Commun..

[45] Hirata J., Kikuchi Y., Imaizumi E., Tode T., Nagata I. (1994). Endometriotic Tissues Produce Immunosuppressive Factors. Gynecol. Obstet. Investig..

[46] Adamson G.D., Pasta D. (2010). Endometriosis fertility index: The new, validated endometriosis staging system. Fertil. Steril..

[47] Catalini L., Fedder J. (2020). Characteristics of the endometrium in menstruating species: Lessons learned from the animal kingdom. Biol. Reprod..

[48] D’Hooghe T., Kyama C., Chai D., Fassbender A., Vodolazkaia A., Bokor A., Mwenda J. (2009). Nonhuman Primate Models for Translational Research in Endometriosis. Reprod. Sci..

[49] Nishimoto-Kakiuchi A., Netsu S., Okabayashi S., Taniguchi K., Tanimura H., Kato A., Suzuki M., Sankai T., Konno R. (2018). Spontaneous endometriosis in cynomolgus monkeys as a clinically relevant experimental model. Hum. Reprod..

[50] D’Hooghe T.M., Bambra C.S., Raeymaekers B.M., De Jonge I., A Hill J., Koninckx P.R. (1995). The effects of immunosuppression on development and progression of endometriosis in baboons (Papio anubis). Fertil. Steril..

[51] Kim C.-H., Chae H.-D., Kang B.-M., Chang Y.S., Mok J.-E. (1997). The Immunotherapy duringin vitroFertilization and Embryo Transfer Cycles in Infertile Patients with Endometriosis. J. Obstet. Gynaecol. Res..

[52] Jerzak M., Niemiec T., Nowakowska A., Klochowicz M., Górski A., Baranowski W. (2010). First Successful Pregnancy after Addition of Enoxaparin to Sildenafil and Etanercept Immunotherapy in Woman with Fifteen Failed IVF Cycles-Case Report. Am. J. Reprod. Immunol..

[53] Önalan G., Tohma Y.A., Zeyneloğlu H.B. (2018). Effect of Etanercept on the Success of Assisted Reproductive Technology in Patients with Endometrioma. Gynecol. Obstet. Investig..

[54] Itil I.M., Cirpan T., Akercan F., Gamaa A., Kazandi M., Kazandi A.C., Yildiz P.S., Askar N. (2006). Effect of BCG vaccine on peritoneal endometriotic implants in a rat model of endometriosis. Aust. N. Z. J. Obstet. Gynaecol..

[55] Hecht J., Suliman S., Wegiel B. (2021). Bacillus Calmette–Guerin (BCG) vaccination to treat endometriosis. Vaccine.

[56] Szymanowski K., Chmaj-Wierzchowska K., Yantczenko A., Niepsuj-Biniaś J., Florek E., Opala T., Murawski M. (2009). Endometriosis prophylaxis and treatment with the newly developed xenogenic immunomodulator RESAN in an animal model. Eur. J. Obstet. Gynecol. Reprod. Biol..

[57] Szymanowski K., Niepsuj-Biniaś J., Dera-Szymanowska A., Wolun-Cholewa M., Yantczenko A., Florek E., Opala T., Murawski M., Wiktorowicz K. (2013). An Influence of Immunomodulation on Th1 and Th2 Immune Response in Endometriosis in an Animal Model. BioMed Res. Int..

[58] Ścieżyńska A., Komorowski M., Soszyńska M., Malejczyk J. (2019). NK Cells as Potential Targets for Immunotherapy in Endometriosis. J. Clin. Med..

[59] Li Z., You Y., Griffin N., Feng J., Shan F. (2018). Low-dose naltrexone (LDN): A promising treatment in immune-related diseases and cancer therapy. Int. Immunopharmacol..

[60] McLaughlin P.J., McHugh D.P., Magister M., Zagon I.S. (2015). Endogenous opioid inhibition of proliferation of T and B cell subpopulations in response to immunization for experimental autoimmune encephalomyelitis. BMC Immunol..

[61] Ahmed M., Duleba A., El Shahat O., Ibrahim M., Salem A. (2008). Naltrexone treatment in clomiphene resistant women with polycystic ovary syndrome. Hum. Reprod..

[62] Younger J., Parkitny L., McLain D. (2014). The use of low-dose naltrexone (LDN) as a novel anti-inflammatory treatment for chronic pain. Clin. Rheumatol..

[63] Kaya H., Oral B. (1999). Effect of ovarian involvement on the frequency of luteinized unruptured follicle in endometriosis. Gynecol. Obstet. Investig..

[64] Waseda T., Tomizawa H., Fujii R., Makinoda S., Hirosaki N. (2008). Granulocyte Colony-Stimulating Factor (G-CSF) in the Mechanism of Human Ovulation and its Clinical Usefulness. Curr. Med. Chem..

[65] Izumi G., Koga K., Takamura M., Bo W., Nagai M., Miyashita M., Harada M., Hirata T., Hirota Y., Yoshino O. (2017). Oil-Soluble Contrast Medium (OSCM) for Hysterosalpingography Modulates Dendritic Cell and Regulatory T Cell Profiles in the Peritoneal Cavity: A Possible Mechanism by Which OSCM Enhances Fertility. J. Immunol..

[66] Peart J.M., Sim R.G., Hofman P.L. (2021). Therapeutic effects of hysterosalpingography contrast media in infertile women: What do we know about the H2O in the H2Oil trial and why does it matter?. Hum. Reprod..

[67] Mathews D.M., Johnson N.P., Sim R.G., O’Sullivan S., Peart J.M., Hofman P.L. (2021). Iodine and fertility: Do we know enough?. Hum. Reprod..

[68] Johnson N.P. (2014). Review of lipiodol treatment for infertility—An innovative treatment for endometriosis-related infertility?. Aust. N. Z. J. Obstet. Gynaecol..

[69] Daniilidis A., Pados G. (2018). Comments on the ESHRE recommendations for the treatment of minimal endometriosis in infertile women. Reprod. Biomed. Online.

[70] Johnson N.P., Hummelshoj L., Consortium W.E.S.M., Abrao M., Adamson G., Allaire C., Amelung V., Andersson E., Becker C., Birna Árdal K. (2013). Consensus on current management of endometriosis. Hum. Reprod..

[71] Practice Committee of the American Society for Reproductive Medicine (2012). Endometriosis and infertility: A committee opinion. Fertil. Steril..

